# Lee Silverman Voice Treatment versus standard speech and language therapy versus control in Parkinson’s disease: a pilot randomised controlled trial (PD COMM pilot)

**DOI:** 10.1186/s40814-017-0222-z

**Published:** 2018-01-10

**Authors:** Catherine M. Sackley, Christina H. Smith, Caroline E. Rick, Marian C. Brady, Natalie Ives, Smitaa Patel, Rebecca Woolley, Francis Dowling, Ramilla Patel, Helen Roberts, Sue Jowett, Keith Wheatley, Debbie Kelly, Gina Sands, Carl E. Clarke, on behalf of the PD COMM Pilot Collaborative Group

**Affiliations:** 1 Faculty of Life Sciences and Medicine, King’s College London, London, UK; 2 Division of Psychology and Language Science, Faculty of Brain Sciences, University College London, London, UK; 3 Birmingham Clinical Trials Unit (BCTU), University of Birmingham, Birmingham, UK; 4 Nursing, Midwifery and Allied Health Professions Research Unit, Glasgow Caledonian University, Glasgow, UK; 5 Parkinson’s UK West Midlands Regional Branch, London, UK; 6 Faculty of Medicine, University of Southampton, London, UK; 7 Institute for Applied Health Research, University of Birmingham, Birmingham, UK; 8 Cancer Research UK Clinical Trials Unit (CRCTU), University of Birmingham, Birmingham, UK; 9 School of Rehabilitation Sciences, University of East Anglia, Norwich, UK; 10 Department of Neurology, Clinical Neurology City Hospital, Sandwell and West Birmingham Hospitals NHS Trust, Dudley Road, Birmingham, B18 7QH UK; 11 School of Health Sciences, University of Nottingham, Nottingham, UK

**Keywords:** Parkinson’s disease, Pilot randomised controlled trial, Speech and language therapy

## Abstract

**Background:**

Speech-related problems are common in Parkinson’s disease (PD), but there is little evidence for the effectiveness of standard speech and language therapy (SLT) or Lee Silverman Voice Treatment (LSVT LOUD®).

**Methods:**

The PD COMM pilot was a three-arm, assessor-blinded, randomised controlled trial (RCT) of LSVT LOUD®, SLT and no intervention (1:1:1 ratio) to assess the feasibility and to inform the design of a full-scale RCT. Non-demented patients with idiopathic PD and speech problems and no SLT for speech problems in the past 2 years were eligible. LSVT LOUD® is a standardised regime (16 sessions over 4 weeks). SLT comprised individualised content per local practice (typically weekly sessions for 6–8 weeks). Outcomes included recruitment and retention, treatment adherence, and data completeness. Outcome data collected at baseline, 3, 6, and 12 months included patient-reported voice and quality of life measures, resource use, and assessor-rated speech recordings.

**Results:**

Eighty-nine patients were randomised with 90% in the therapy groups and 100% in the control group completing the trial. The response rate for Voice Handicap Index (VHI) in each arm was ≥ 90% at all time-points. VHI was highly correlated with the other speech-related outcome measures. There was a trend to improvement in VHI with LSVT LOUD® (difference at 3 months compared with control: − 12.5 points; 95% CI − 26.2, 1.2) and SLT (difference at 3 months compared with control: − 9.8 points; 95% CI − 23.2, 3.7) which needs to be confirmed in an adequately powered trial.

**Conclusion:**

Randomisation to a three-arm trial of speech therapy including a no intervention control is feasible and acceptable. Compliance with both interventions was good. VHI and other patient-reported outcomes were relevant measures and provided data to inform the sample size for a substantive trial.

**Trial registration:**

International Standard Randomised Controlled Trial Number Register: ISRCTN75223808. registered 22 March 2012.

## Background

Speech problems affect 51–74% of patients with Parkinson’s disease (PD). [1–3] Speech changes can occur in the early stages of the disease and difficulty in communication can lead to social isolation. In a UK survey of 125 people with mainly early PD, Miller and colleagues [4] found only 4.2% reported no changes to their speech or voice and 82% were dissatisfied with how they spoke. For 10%, speech was their main concern amongst all the changes experienced due to PD and 38% placed speech in their top four concerns.

Drug therapy has only modest effects on prosodic aspects of parkinsonian speech, so other therapeutic measures such as speech and language therapy (SLT) could play a role in treatment. [5] Conventional SLT is tailored to individual patients’ needs and may include: diaphragmatic breathing, pacing/rate control, word-finding strategies, and voice/articulation exercises. [6] One technique that has been used in PD is attention to effort, where the speaker is asked to produce a loud voice and focus their effort on attaining, monitoring, and maintaining this. This technique was formalised in an evidence-based commercially available programme provided by the Lee Silverman Voice Treatment (LSVT LOUD®) organisation from the late 1980s. [7] Several studies [8–11] showed promising results of LSVT LOUD® producing, not only louder voice, but also gains in articulatory parameters which were sustained at follow-up. Cochrane reviews have summarised the evidence for the efficacy of various forms of SLT in PD, but concluded that there was insufficient evidence to support the use of one form of SLT over another and recommended a large, methodologically sound randomised controlled trial (RCT), with follow-up of at least 6 months and meaningful outcome measures. [12, 13]

The PD COMM pilot trial assessed the feasibility and acceptability of a large-scale RCT to assess the clinical and cost effectiveness of LSVT LOUD® versus standard SLT versus no intervention in dysarthria associated with PD. In accordance with guidance of the Medical Research Council (MRC) for trials of complex interventions [14] the following parameters were assessed: (1) feasibility and acceptability of randomising PD patients with problems of speech or voice to LSVT LOUD®, traditional SLT interventions or no intervention control; (2) patient eligibility, recruitment, and retention rates; (3) numbers of sites and patients that need to be screened; (4) time required to undertake a full-scale trial; (5) acceptability and adherence with LSVT LOUD®; (6) dose and content of traditional SLT; (7) data completeness and suitability of data collection methods; (8) assessment of the most suitable primary outcome measure for the full-scale trial and to obtain initial estimates to inform the sample size calculation; and (9) pilot bespoke and standard health economic evaluation questionnaires.

## Methods

### Study design

The PD COMM pilot trial protocol has been published. [15] The trial was designed as a multicentre three-arm parallel group randomised controlled pilot trial with blinded assessor. It was sponsored by the University of Birmingham, received ethical approval from the West Midlands, Coventry and Warwick NHS Research Ethics Committee (11/WM/0343), and local NHS R&D approval at each site prior to the start of recruitment. The trial was managed by the University of Birmingham Clinical Trials Unit (BCTU). Due to the nature of the intervention, therapists and patients were not blinded to treatment allocation; however, assessors of the vocal assessment outcome data were all blinded to treatment allocation for the duration of the trial.

### Patients

Eligibility criteria were idiopathic PD defined by the UK Parkinson’s Disease Society Brain Bank Criteria; [16] and presence of patient or carer-reported problems with speech. [1] Exclusion criteria were dementia as defined clinically by the physician; evidence of laryngeal pathology including vocal nodules, a history of vocal strain, or previous laryngeal surgery as LSVT LOUD® is not appropriate for all of this group; [9] received SLT for PD speech-related problems in the past 2 years; and the investigator thought that the patient did not definitely require SLT in the short term.

### Consent and randomisation

Potential patients who met the eligibility criteria were approached in their normal outpatient appointments. If interested, they were given a patient information sheet and time to consider the trial and discuss it with friends and family. Following consent, patients completed baseline assessments prior to randomisation. For practical reasons, baseline vocal assessments were allowed to be performed after randomisation, but had to be completed prior to the start of therapy.

After completing the baseline questionnaires, patients were randomised in a 1:1:1 ratio to the three groups via the trials unit telephone randomisation service. This secure central randomisation service was available from 9 am to 5 pm weekdays and ensured the concealment of treatment allocation. A computer-generated randomisation list was used. Patients and therapists were informed of the treatment allocation, but assessors of the vocal assessments remained blind to treatment allocation. If allocated to an intervention arm, referral to the appropriate speech and language therapist occurred immediately following randomisation. All personal information obtained for the study was held securely and treated as strictly confidential.

### Interventions

Both SLT and LSVT LOUD® were delivered either in community-based healthcare places or in outpatient neurology units in the UK.

LSVT LOUD® was administered in four sessions per week for 4 weeks (i.e. 16 sessions in total) by state registered speech and language therapists with certification in Lee Silverman Voice Therapy and appropriate refresher courses working within the NHS. Each session lasted 50–60 min. In addition, patients were asked to complete 5–10 min of home practise on treatment days and up to 30 min of home practise on non-treatment days. LSVT LOUD® comprises maximum effort non-speech and speech drills. The non-speech drills include production of sustained ‘ah’ phonation at a single pitch and pitch glides (moving from modal pitch to high pitch and modal pitch and going down on production of sustained ‘ah’). These exercises are for improving vocal effort and loudness for translation into functional speech. The speech drills utilise a hierarchy of speech tasks moving from single words through phrases and onto conversational speech. Each step in this hierarchy puts increased demands on the speaker and challenges the speaker to maintain maximal speech production. It is important to note the intervention incorporates retraining the sensory system to improve loudness.

SLT was administered as per local practice by state-registered speech and language therapists and was expected to typically involve one session of 45 min per week for 6–8 weeks of varying content as determined by patient need. Treatments could include exercises targeting respiration, phonation, articulation [17, 18], behavioural strategies to reduce prosodic abnormality [19], and the use of augmentative and alternative communication (AAC) strategies and therapeutic devices to improve functional communication [20].

Those individuals allocated to the control group continued with their standard PD care. They were excluded from receiving SLT input for at least 6 months post-randomisation, unless their clinician deemed it to be medically necessary. After 6 months, people in the control arm were eligible to be referred for therapy if required.

Training on trial processes was provided for trial therapists by the clinical trial team to ensure uniformity of trial procedures. Therapists providing the interventions completed intervention record forms at each session, as used in previous complex intervention trials, [21] to allow monitoring of intervention delivery.

### Sample size

As this was a pilot study, no formal sample size calculation was performed. The study aimed to recruit at least 20 patients in each group, a total of at least 60 patients.

### Outcomes

Data on various outcome measures were collected to assess appropriate outcome measures to be used in a large-scale trial: patient-reported measures-Voice Handicap Index (VHI) [22], Parkinson’s Disease Questionnaire-39 (PDQ-39) [23], voice-related quality of life scale (V-RQoL) [24], Living with Dysarthria questionnaire (LwD) [25], EuroQol (EQ-5D) [26, 27], ICECAP capability measure for older people (ICECAP-O) [28], and resource usage; therapist measures-vocal loudness, comprehension assessments, and Assessment of Intelligibility of Dysarthric Speech (AIDS) [29]; carer-reported quality of life (Parkinson’s Disease Questionnaire–Carer, PDQ-Carer [30]); and adverse events. The questionnaires used were all validated and widely used tools. Data were collected before randomisation and 3, 6, and 12 months after randomisation. The bespoke resource usage questionnaire was assessed at 3, 6, and 12 months for suitability in a definitive trial. This included questions on health and social care resource use, employment and time off work, and out of pocket costs incurred by patients.

Following a risk assessment, it was agreed that this was a low risk trial and that only vocal strain or abuse were likely to be related to the interventions. Therefore, targeted treatment-related adverse events and serious adverse events such as vocal strain or abuse were collected.

### Data analyses

As this was a feasibility study, definitive comparisons of the interventions were not undertaken. Feasibility measures and outcome data were therefore summarised descriptively. Details on patient screening, recruitment and retention, withdrawals and those lost to follow-up, along with reasons for non-completion, and adherence were summarised using a CONSORT diagram (objectives 1–5). Adherence with LSVT LOUD was assessed as the proportion of patients who completed the intervention as per the protocol (objective 5). Information on the interventions including the median number and mean duration of sessions was summarised descriptively (objectives 5 and 6). The percentage of forms returned and level of data completeness at each time point was tabulated (objective 7). Assessment of the most suitable primary outcome measure for the full-scale trial and which outcomes to retain in a substantive trial included (1) an assessment of data completeness and (2) correlation methods to identify which outcome measures were closely correlated (objective 8). To help inform the sample size calculation, the mean and standard deviation for each outcome was summarised at each time point and an exploratory analysis of differences between the arms (LSVT LOUD® versus no intervention, SLT versus no intervention, and LSVT LOUD® versus SLT) was performed, calculating the mean difference at each time point and the mean change from baseline to 3, 6, and 12 months alongside 95% confidence intervals (CI) (objective 8). Missing values in PDQ-39 domain scores were imputed using an expectation maximisation algorithm.[31, 32] As is standard for phase III clinical trials, the pilot outcome data were analysed using intention-to-treat methods with patients analysed in the treatment group to which they were randomised regardless of adherence to the intervention or protocol. Statistical analysis was performed using SAS version 9.4 software.

## Results

### Patient acceptability, screening, recruitment, and retention (objectives 1–4)

Sites reported screening 2223 patients, with 89 patients randomised into the PD COMM pilot trial from 12 centres between May 2012 and March 2014. Data on the potential participants screened showed variations in recruitment methods: some centres screened PD clinic populations sequentially, whereas others recruited patients from therapist services. The reasons patients were not entered into the trial were no problems with speech or voice (*n* = 1406), had SLT or likely to (*n* = 177), dementia (*n* = 176), too unwell (*n* = 119), already in a trial (*n* = 92), very little English (*n* = 21), and declined (*n* = 143). Therefore, the main reason for non-entry into the trial was that the patient was not eligible (79%). Only 6% of screened participants declined the trial which suggests that the study was acceptable to patients.

**Fig. 1:**
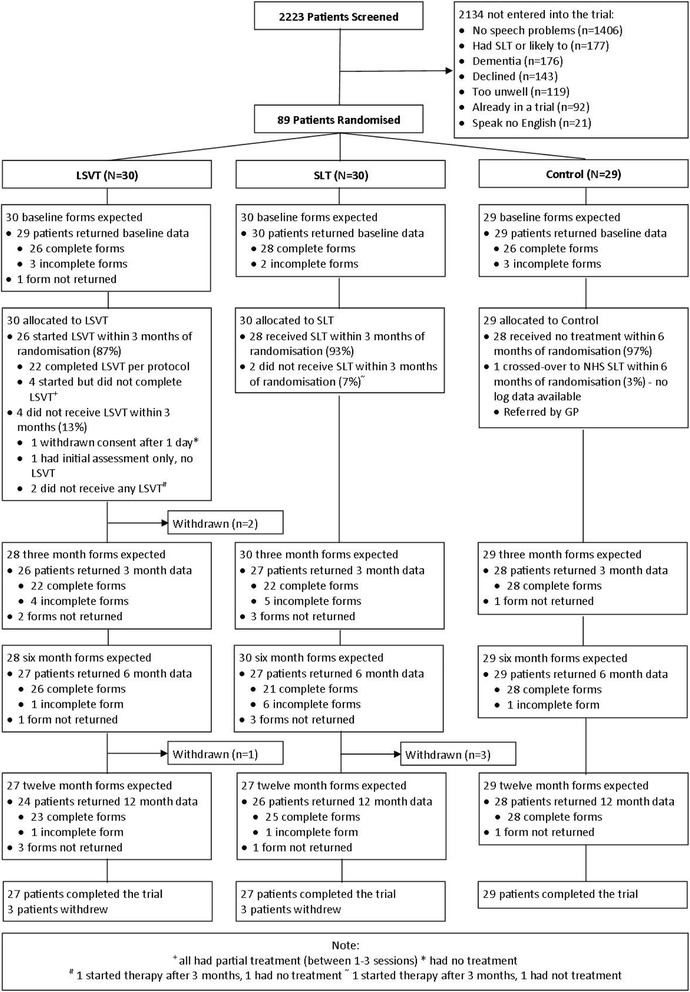
Flow of patients through the trial

Thirty patients were randomised to LSVT LOUD®, 30 to SLT, and 29 to the control group, with 27 (90%), 27 (90%), and 29 (100%) completing the trial, respectively (Fig. 1).

### Treatment fidelity, adherence, and content (objectives 5 and 6)

In the LSVT LOUD® group, 26 of 30 patients started LSVT, with 22 (73%) completing LSVT as per protocol (Fig. 1). Seven patients randomised to LSVT LOUD® either did not start (*n* = 3) or stopped therapy early (i.e. did not complete 16 sessions; *n* = 4). The four patients that stopped therapy received 1–3 sessions. Three of these seven patients withdrew from the trial citing the intensity and time commitment of LSVT LOUD® as the reason for withdrawal (Fig. 1). One patient randomised to SLT did not start therapy for family reasons and then withdrew from the trial.

In the LSVT LOUD® group, 47% of patients had their initial interview within 4 weeks of randomisation compared to 57% in the standard SLT group. Delivery of the intervention was good, with 96% in the LSVT LOUD® arm and 97% in the SLT arm starting treatment within 3 months, and 86% in the LSVT LOUD® arm and 73% in the SLT arm completing treatment within 3 months. In the LSVT LOUD® group, patients had a median of 16 sessions lasting on average 61 min over 4.7 weeks. In the SLT group, patients had a median of 6 sessions lasting on average 54 min over 9.6 weeks.

### Form return rates and data completeness (objective 7)

Data return rates were very good (> 90%). The combined response rates for VHI was 99, 93, 95, and 94% at baseline, 3, 6, and 12 months follow-up, respectively, and the return rates were balanced across the arms. Completion of the VHI forms was close to the planned time points, and data completeness was good. Similar return rates and levels of data completeness were seen across all the other outcome measures. In the V-RQoL, one question was not answered reliably (“I have trouble doing my job or practising my profession*”*), so this questionnaire has been dropped in the main trial.

### Patient characteristics at randomisation

Patients entering the trial had a mean age of 67 years (male 78%; body mass index 27 kg/m^2^). Mean disease duration was 5.5 years with a baseline Hoehn and Yahr stage ≤ 2.0 in 66%. The mean baseline levodopa dose equivalent was 580 mg/day (Table 1). [33] Thirty-five patients had a regular carer of which 29 (83%) consented to enter the trial and complete the PDQ-Carer questionnaire (13 LSVT, 11 NHS, 5 control). Most carers were female and spouses.

**Table 1:** Patient characteristics at randomisation

		LSVT	SLT	Control
Number of patients randomised		30	30	29
Age (years)	Mean (SD)	67 (8.4)	68 (10.3)	65 (7.5)
Gender	Male (N, %)	23 (77%)	23 (77%)	23 (79%)
Body Mass Index (kg/m^2^)	Mean (SD)	27.4 (4.3)	27.6 (4.8)	27.3 (4.2)
Duration of PD (years)	Mean (SD)	6.1 (3.7)	5.6 (4.2)	4.9 (3.4)
Hoehn and Yahr stage	≤ 2.0	20 (67%)	16 (55%)	20 (77%)
	2.5	5 (17%)	2 (7%)	5 (19%)
	3.0	4 (13%)	9 (31%)	1 (4%)
	≥ 4.0	1 (3%)	2 (7%)	0 (−)
Levodopa equivalent dose (mg/day)^a^	Mean (SD)	695 (466.4)	533 (328.5)	502 (451.6)

^a^Levodopa equivalency formula from reference [33]

### Assessment of outcome measures for the full-scale trial (objective 8)

Correlations between the patient and therapist-assessed outcomes were varied (Table 2; range − 0.58 to 0.02), but patient-reported outcomes correlated well with each other (*r* > 0.7). Interestingly, vocal loudness did not correlate well with the patient-reported measures (*r* < 0.2).

**Table 2:** Pearson correlation coefficients of participant and therapist-rated outcomes

	Baseline				3 months			
	VHI–total score	PDQ-39 communication	V-RQoL	LwD	VHI	PDQ-39 communication	V-RQoL	LwD
Participant-rated								
VHI-total score	1.00	–	–	–	1.00	–	–	–
PDQ-39 communication	0.73	1.00	–	–	0.74	1.00	–	–
V-RQoL	0.86	0.76	1.00	–	0.90	0.77	1.00	–
LwD	0.77	0.73	0.78	1.00	0.78	0.75	0.79	1.00
Therapist-rated								
AIDS words	−0.64	−0.45	−0.46	−0.33	−0.53	−0.30	−0.31	−0.38
AIDS sentences	−0.65	−0.43	−0.45	−0.35	−0.58	−0.34	−0.36	−0.35
Rainbow passage	0.17	0.15	0.16	0.08	−0.11	0.02	−0.15	−0.12
Cookie theft	−0.10	0.03	0.03	−0.14	−0.14	−0.02	−0.12	−0.18
Vocal loudness	−0.09	−0.15	−0.06	−0.12	−0.16	−0.10	−0.12	−0.18

*VHI* Voice Handicap Index, *PDQ-39* Parkinson’s Disease Questionnaire-39, *V-RQoL* voice-related quality of life score, *LwD* Living with Dysarthria score

In a survey of patients with PD from our Patient and Public Involvement Group, we asked patients what was more important to them: vocal loudness or ability to communicate. The results showed that although vocal loudness was important, it was only one aspect of a complex problem which was also influenced by environmental factors (e.g. dry mouth, stress levels), and that patients preferred a more generic overall assessment of voice problems.

Since VHI correlated best with therapist-assessed outcomes and the PDQ-39 is a well-validated questionnaire used in PD research, we investigated both the VHI total score and PDQ-39 communication domain further as possible primary outcome measures for the main trial.

### Data to inform the sample size calculation (objective 8)

**Fig. 2:**
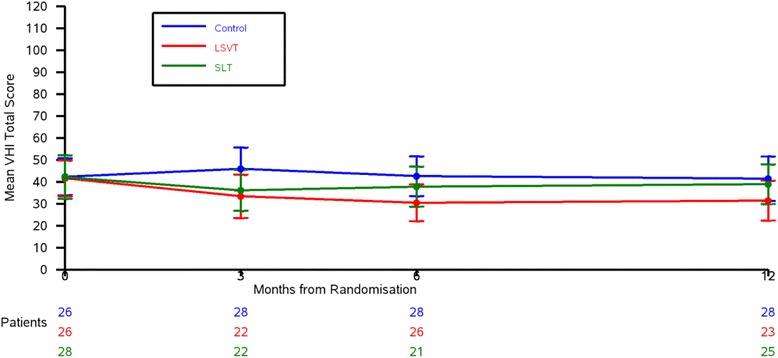
VHI total score over time

**Fig. 3:**
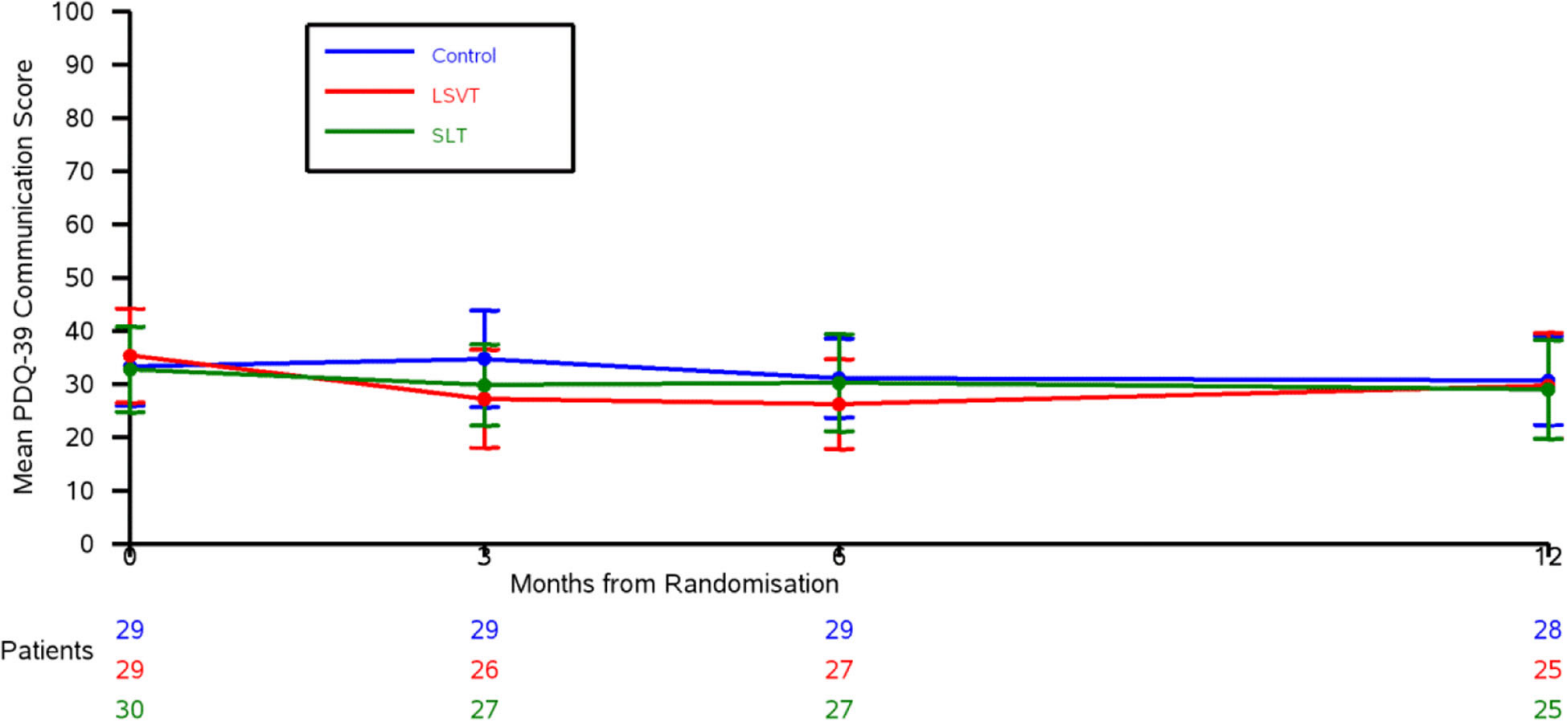
PDQ-39 communication domain over time

There was a − 12.5-point difference (95% CI − 26.2, 1.2) in the VHI total score at 3 months between LSVT LOUD® and control group, and a difference of − 9.8 points (95% CI − 23.2, 3.7) in the VHI total score at 3 months between SLT and control group. For the PDQ-39 communication score, at 3 months, there was a 7.5-point difference (95% CI − 20.3, 5.2) between the LSVT LOUD® and control group (Table 3), and a 5.0-point difference (95% CI − 16.7 to 6.8) between the SLT and control groups. The VHI total score and PDQ-39 communication domain data at baseline, and 3, 6, and 12 months are shown in Figs. 2 and 3.

**Table 3:** Results of participant-rated and carer-rated outcomes

	Baseline			3 Months				
	LSVT	NHS	Control	LSVT	NHS	Control	LSVT vs. control	NHS vs. control
VHI total score	*N* = 26 42 (20.2)	*N* = 28 42 (25.5)	*N* = 26 42 (21.0)	*N* = 22 33 (22.4)	*N* = 22 36 (21.2)	*N* = 28 46 (25.1)	− 12.5 (− 26.2 to 1.2)	− 9.8 (− 23.2 to 3.7)
PDQ-39 communication domain	*N* = 29 35 (23.3)	*N* = 30 33 (21.5)	*N* = 29 33 (19.5)	*N* = 26 27 (22.9)	*N* = 27 30 (19.4)	*N* = 29 35 (24.0)	− 7.5 (− 20.3 to 5.2)	− 5.0 (− 16.7 to 6.8)
PDQ-39 summary index	*N* = 29 32 (15.5)	*N* = 30 28 (13.8)	*N* = 29 26 (14.1)	*N* = 26 29 (17.5)	*N* = 27 27 (13.8)	*N* = 29 29 (16.3)	− 0.2 (− 9.3 to 9.0)	− 2.1 (− 10.2 to 6.0)
V-RQoL	*N* = 27 20 (8.9)	*N* = 25 20 (8.3)	*N* = 25 21 (7.1)	*N* = 21 18 (7.8)	*N* = 24 19 (5.6)	*N* = 28 22 (8.0)	− 3.5 (− 8.1 to 1.1)	− 3.2 (− 7.1 to 0.7)
LwD	*N* = 27 28 (16.2)	*N* = 27 32 (21.9)	*N* = 26 27 (20.7)	*N* = 25 24 (21.6)	*N* = 24 28 (17.1)	*N* = 25 29 (20.4)	− 5.6 (− 17.6 to 6.3)	− 1.9 (− 12.8 to 8.9)
EQ-5D QoL score	*N* = 29 0.59 (0.30)	*N* = 30 0.64 (0.23)	*N* = 29 0.72 (0.18)	*N* = 26 0.60 (0.27)	*N* = 27 0.70 (0.20)	*N* = 28 0.60 (0.29)	0.004 (− 0.15 to 0.16)	0.11 (− 0.03 to 0.25)
PDQ-Carer summary index	*N* = 11 27 (19.7)	*N* = 11 26 (20.9)	*N* = 3 15 (18.2)	*N* = 11 32 (22.9)	*N* = 7 21 (13.4)	*N* = 4 18 (17.8)	15.8 (− 15.7 to 47.3)	5.9 (− 19.1 to 30.9)

Mean difference (95% CI) for comparisons

VHI ranges from 0 to 120; PDQ-39 ranges from 0 to 100; V-RQoL ranges from 10 to 50; LwD ranges from 0 to 90; PDQ-Carer ranges from 0 to 100, where low score is good. Negative difference favours treatment

EQ-5D ranges from − 0.59 to 1, where high score is good. Positive difference favours treatment

The minimum clinically important change (MCIC) for the communication domain of the PDQ-39 is 4.2 points [34]. The mean baseline score was 33.8, assuming no deterioration in the control arm, the MCIC corresponds to detecting a conservative 12% difference and a small effect size of 0.17 SD. The differences between LSVT LOUD® and SLT versus control at 3 months were 7.5 and 5.0 points, respectively. Although these differences are greater than the MCIC (4.2 points), they are close enough to the MCIC to make it difficult to justify powering a definitive study on a larger difference than the MCIC. If we used the PDQ-39 communication as our primary outcome, at 80% power and using α = 0.025 (to adjust for multiple comparisons), we would need to recruit 2028 patients to detect a difference of 4.2 points (the MCIC), which is unfeasible. The MCIC for VHI has not been established for this cohort of patients. The differences in VHI total score at 3 months between LSVT LOUD® and SLT versus control were 12.5 and 9.8 points, respectively. Assuming a difference of 10 points between therapy groups and control, along with the upper standard deviation of 26.3, the effect size is moderate at 0.38 SD. Due to the nature and cost of the interventions, and the trial primarily comparing intervention versus control, this justifies investigating a moderate effect size. The VHI is also a questionnaire that specifically asks an individual to describe their voice and the effects of their voice on their life. We therefore chose the VHI total score as the primary outcome measure for the substantive trial. To detect a 10-point difference in VHI total score at 3 months (upper SD 26.3; 80% power; α = 0.025) requires 399 patients (133 per group).

### Safety

There were no adverse events or serious adverse events reported in the trial.

### Pilot health economic evaluation questionnaires (objective 9)

A substantive trial should also contain a full economic evaluation to estimate the incremental cost-effectiveness of the LSVT LOUD® intervention versus SLT and no intervention. Piloting the bespoke resource use questionnaire demonstrated that it was suitable, as the completion rate was good. The EQ-5D and the ICECAP-O were confirmed to be suitable economic outcome measures, to measure both health-related quality of life and broader aspects of capability.

## Discussion

The results of the PD COMM pilot study have shown that a large-scale trial to evaluate the efficacy and cost-effectiveness of Lee Silverman Voice Treatment versus standard NHS speech and language therapy versus control for communication problems in PD is both acceptable and feasible. The UK Medical Research Council advises that in feasibility trials of complex interventions a number of parameters should be assessed which we discuss in the following paragraphs. [14]

We originally aimed to recruit 60 patients from four centres over 18 months, but expanded to 12 centres because of slow recruitment, eventually recruiting 89 patients over 23 months.

The main reasons potential participants did not take part in the study were lack of speech problems (66%), dementia (8%), and declined consent (7%). The discrepancy between the estimates of prevalence of problems and those in the recruiting NHS clinics is interesting and important for planning the full trial, however, it is unexplained at present.

Randomisation to a no treatment arm was acceptable to patients and clinicians, and retention rates during the whole trial were high at around 90%. There was a concern that the high intensity of LSVT LOUD® might lead to a high withdrawal rate. A number of patients decided not to enter the trial because of the intensity of LSVT LOUD® which did affect recruitment rates. Of those who entered the trial, seven in the LSVT LOUD® arm either did not start therapy or stopped LSVT LOUD® early, with three of these patients withdrawing from the trial. This compares with only one patient in the SLT arm who did not start therapy.

Our study has demonstrated the ability to successfully deliver two distinct complex SLT interventions for dysarthria associated with PD which differed in session length, time to intervention, overall dose of therapy, and intervention duration. High intensity SLT therapy is not tolerated by all patients, and the results for trials employing such approaches amongst other patient groups have been confounded by significantly higher dropout rates (than seen in our study) from the high intensity groups [35]. Intervention delivery will be a challenging issue during the substantive trial, particularly given the difficult financial situation within the National Health Service. However, delivery of the intervention in the pilot was good, with most patients starting and completing the intervention within 3 months of randomisation. It was noted that there was a slight difference in the number of patients completing treatment by 3 months (86% in the LSVT LOUD® group compared with 73% in the SLT group); we will monitor this closely within the main trial.

A battery of patient and carer reported assessments were employed in the study to evaluate the feasibility, acceptability, sensitivity, and correlation of outcome measures. Data return and completeness for all outcome measures at each time point was excellent. Correlations between the patient and therapist-assessed outcomes were varied, but patient-reported outcomes correlated well with each other (*r* > 0.7). Vocal loudness did not correlate well with patient-reported measures (*r* < 0.2). Previous trials have used vocal loudness as the primary outcome measure, but it is not clear whether this captures what is important to patients in terms of communication. Our survey of a number of patients with PD showed that patients preferred and wanted a more generic overall assessment of voice problems.

Since the VHI correlated best with the therapist-assessed outcomes and the PDQ-39 is a well-validated questionnaire used in PD research, we investigated both the VHI total score and PDQ-39 communication domain as possible primary outcome measures for the main trial. The sample sizes for a full-scale trial using these outcomes (with 80% power, α = 0.025 (to adjust for multiple comparisons)) were 2028 patients with the PDQ-39 communication domain and 399 patients with the VHI total score. A 2000 patient trial was not feasible, and based on the VHI asking an individual to describe their voice and the effects of their voice on their life, which came out as important from our patient survey, the VHI total score was chosen as the primary outcome. To detect a 10-point difference in VHI total score at 3 months (upper SD 26.3; 80% power; α = 0.025) will require 399 patients (133 per arm). To allow for 10% drop out, a total of 450 patients (150 per arm) will be recruited. From the feasibility study, six patients can be recruited per site per year, so with 40 sites, 450 patients can be recruited in just under 2 years.

## Conclusions

PD COMM pilot is the largest trial to date of SLT in PD. The three trials in the Cochrane review included a total of only 63 patients [13] and the most recent trial of LSVT LOUD® LOUD and ARTIC versus no therapy included only 64 patients. [36] The PD COMM pilot trial demonstrated that both LSVT LOUD® and SLT may be effective in improving communication in PD, although this needs to be confirmed in an adequately powered trial. Our study established that such a substantive trial is both feasible and acceptable to PD patients and therapists treating their communication problems. A large-scale trial (PD COMM) is now underway in the United Kingdom.

## Abbreviations

AAC: Augmentative and alternative communication strategies
AIDS: Assessment of Intelligibility of Dysarthric Speech
BCTU: University of Birmingham Clinical Trials Unit
CI: Confidence intervals
EQ-5D: EuroQoL
ICECAP-O: ICECAP capability measure for older people
LSVT LOUD®: Lee Silverman Voice Treatment
LwD: Living with Dysarthria questionnaire
MCIC: Minimum clinically important change
MRC: Medical Research Council
PD: Parkinson’s disease
PDQ-39: Parkinson’s Disease Questionnaire-39
PDQ-Carer: Parkinson’s Disease Questionnaire-Carer
RCT: Randomised controlled trial
SLT: Standard speech and language therapy
VHI: Voice Handicap Index
V-RQoL: Voice-related quality of life scale
